# A Statistical Method and Tool to Account for Indirect Calorimetry Differential Measurement Error in a Single-Subject Analysis

**DOI:** 10.3389/fphys.2016.00172

**Published:** 2016-05-11

**Authors:** Matthew S. Tenan

**Affiliations:** United States Army Research Laboratory, Human Research and Engineering Directorate, Integrated Capability Enhancement Branch, Aberdeen Proving GroundAberdeen, MD, USA

**Keywords:** exercise testing, VO_2_, indirect calorimetry, research methods, cost of transport

## Abstract

Indirect calorimetry and oxygen consumption (VO_2_) are accepted tools in human physiology research. It has been shown that indirect calorimetry systems exhibit differential measurement error, where the error of a device is systematically different depending on the volume of gas flow. Moreover, systems commonly report multiple decimal places of precision, giving the clinician a false sense of device accuracy. The purpose of this manuscript is to demonstrate the use of a novel statistical tool which models the reliability of two specific indirect calorimetry systems, Douglas bag and Parvomedics 2400 TrueOne, as univariate normal distributions and implements the distribution overlapping coefficient to determine the likelihood that two VO_2_ measures are the same. A command line implementation of the tool is available for the R programming language as well as a web-based graphical user interface (GUI). This tool is valuable for clinicians performing a single-subject analysis as well as researchers interested in determining if their observed differences exceed the error of the device.

## Introduction

Since the original description of gas exchange indirect calorimetry (Atwater and Benedict, 1983) and progression toward mobility with the Douglas Bag method (Douglas, 1911), the measurement of ventilatory gases has been a mainstay methodology in the field of human physiology. Indirect calorimetry is commonly used to examine the metabolic cost of performing different tasks or to examine the effectiveness of a chronic exercise intervention on cardiovascular fitness. Both of these types of studies share a study design whereby a “baseline” test is performed and a second “experimental” test follows. This test-retest design is common in the area of exercise physiology.

A number of valuable methods have been proposed to examine and understand the effect of measurement error in exercise sciences and these methods can be applied to indirect calorimetry. William Hopkins has developed an ecosystem of tools to understand how reliability alters the understanding of measurements and noise (Hopkins, 2004, 2015). In the case of a test-retest design, the method requires the researcher input measured value, the standard error of measurement or the coefficient of variation, which is the standard error of measurement expressed as a percent of the mean. The Hecksteden et al. method (Hecksteden et al., 2015) uses a similar framework as those proposed by Hopkins (2004, 2015), requiring a measurement and coefficient of variation for the measure. Both of these proposed methodologies are helpful in characterizing test-retest differences for single subjects, and generally assume that measurement error is constant. These methods assume a classical model of non-differential measurement error:

$$\begin{array}{l} W\;=\;X\;+\;U \end{array}$$

In this model, W is the observed value of the mis-measured variable. X is the true variable measured, subject to error and U is the error which is assumed to be independent of X. In the present case, X is the actual VO_2_ (variable of interest) and W is the VO_2_ level actually measured by the device or system.

It is known that error in indirect calorimetry is not constant and has a non-linear measurement error based largely on the total flow rate (Macfarlane and Wu, 2013). This non-random change in measurement error is commonly called “differential measurement error” in epidemiology (Carroll, 2005). In this case the model of differential measurement error will take the general form of:

$$\begin{array}{l} W\;=\;X\;+\;\left(X*U_{X}\right) \end{array}$$

In this model, the error term is not independent of X and may be a linear or non-linear function based upon the value of X. The development of inferential statistical methods where differential measurement error is known are currently under development (Newton et al., 2001; Imai and Yamamoto, 2010). A simpler issue concerns the interpretation of test-retest VO_2_ measures when performing a single subject analysis, which is highly applicable to clinical areas of sport performance and cardiac rehabilitation.

The goal of the present manuscript is to detail the use of a statistical package that models the test-retest reliability of indirect calorimetry as univariate normal distributions accounting for non-linear measurement error. This tool is designed to provide researchers and clinicians a way of determining if two indirect calorimetry measures are likely to be “the same.” The utility of this novel statistical package will be detailed using five hypothetical examples: (1) baseline VO_2_ 1.5 L/min vs. post-intervention VO_2_ 1.7 L/min using the Parvomedics 2400 TrueOne, (2) baseline VO_2_ 3.3 L/min vs. post-intervention VO_2_ 3.5 L/min using the Parvomedics 2400 TrueOne, (3) baseline VO_2_ 1.5 L/min vs. post-intervention VO_2_ 1.7 L/min using the Douglas bag, (4) baseline VO_2_ 3.3 L/min vs. post-intervention VO_2_ 3.5 L/min using the Douglas bag, and (5) baseline VO_2_ 3.0 with the Douglas bag vs. post-intervention VO_2_ 3.3 with the Parvomedics 2400 TrueOne. The proposed tool has both advantages and disadvantages compared to previously proposed methodologies and these differences in both approach and use will be discussed.

## Materials and methods

### Gas.Sim package

Gas.Sim is written in the R programming and statistics language (R Core Team, 2015) which implements “packages” to enhance the capabilities of the base system. The function within the Gas.Sim package for VO_2_ measurement error is called VO2sim. Throughout the package, dplyr is leveraged for data management (Wickham and Francois, 2015) and figures are created using ggplot2 (Wickham, 2015). The graphical user interface (GUI) is created using Shiny for R.

#### Defining the test-retest distribution and overlap

The error around each VO_2_ measurement is modeled as a univariate normal distribution. The parameters for the univariate normal distribution are defined by an analysis performed on the raw data contributed by Crouter et al. (2006). In the study by Crouter et al. (2006), subjects' VO_2_ was measured at increasing cycling workloads on differing days. This provides a range of day-to-day VO_2_ values to create a regression equation modeling the VO_2_ repeatability at different flow rates. The day-to-day variability for the ParvoMedics and Douglas bag methods were determined via identical methodologies. The mean and standard deviation of the two test-retest values were calculated. The data were then fit with a third-order polynomial regression where mean VO_2_ was used to predict the standard deviation of VO_2_ measures. The user-supplied VO_2_ value (*mu*) is combined with the non-linear regression equation to define σ and create the normal distribution density for that measure. When passing two VO_2_ value arguments to VO2sim, two different univariate normal probability distributions are created. All VO_2_ data is input in L/min as this is typically the most “raw” and un-normalized form of the data produced by indirect calorimetry.

The two distributions are next overlapped and the overlapping coefficient is calculated (Inman and Bradley, 1989). The overlapping coefficient is a measure of similarity between two probability distributions and is bounded from 0 to unity (i.e., 1); therefore, the coefficient can be interpreted as a probability that a value obtained in one distribution can also be obtained in the other distribution (i.e., the probability that the same VO_2_ measure is obtained from both distributions).

Gas.Sim presently has one primary function which implements the described analysis for VO_2_ data: VO2_sim. This function is implemented in R and is available upon request from the corresponding author. The function takes 5 inputs:

$$\begin{array}{l} \text{VO2\_sim}(\text{a, b, system\_a}=‘\text{parvo\_2400}’\text{,}\\ \text{system\_b}=‘\text{parvo\_2400}’\text{, plot}=\text{FALSE}) \end{array}$$

The “a” and “b” arguments are the VO_2_ values being tested. The present iteration of VO2sim is valid for use with the ParvoMedics 2400 TrueOne system and Douglas bag, which can be specified with either “parvo_2400” or “douglas_bag,” respectively. The system used to obtain each VO_2_ measure can be specified in the “system_a” or “system_b” argument. In cases where no system is specified, the algorithm defaults to the ParvoMedics 2400 TrueOne system. Depending on the needs of the user, the algorithm can also report only the probability that the two measures are the same (plot=FALSE) or can return a plot of the two distributions with the overlap visually depicted (plot=TRUE); by default, the algorithm simply returns the probability that the two VO_2_ arguments are the same.

#### VO2sim examples

It is pertinent to provide example data to illustrate the utility of the Gas.Sim package. For this purpose, we will examine the effects of theoretical training protocols for persons at a given constant workload. In these examples, repeated VO_2_ measurements will be made with the Douglas bag and with the Parvomedics 2400 TrueOne as well as one example where the baseline data is collected with the Douglas bag but the follow-up test was performed with the Parvomedics 2400 TrueOne. For the lower-end VO_2_ test, the baseline VO_2_ level for both systems is 1.5 L/min. After 1 year of training, the patient/athlete has a VO_2_ of 1.7 L/min, measured with both systems. For the higher-end VO_2_ test, the baseline VO_2_ level is 3.3 L/min and the post-intervention measure is 3.5 L/min. The fifth example assumes the first test was performed with the Douglas bag (VO_2_: 3.0 L/min) and the follow-up test was performed with the Parvomedics 2400 TrueOne (VO_2_: 3.3 L/min). VO2sim will be used to determine the probability that the change in VO_2_ observed for all pre- post-testing arise from the same distribution (i.e., they are the same measurement with no “true” change).

## Results

### Vo2sim: visualizing example distributions and classification

**Figure 1:**
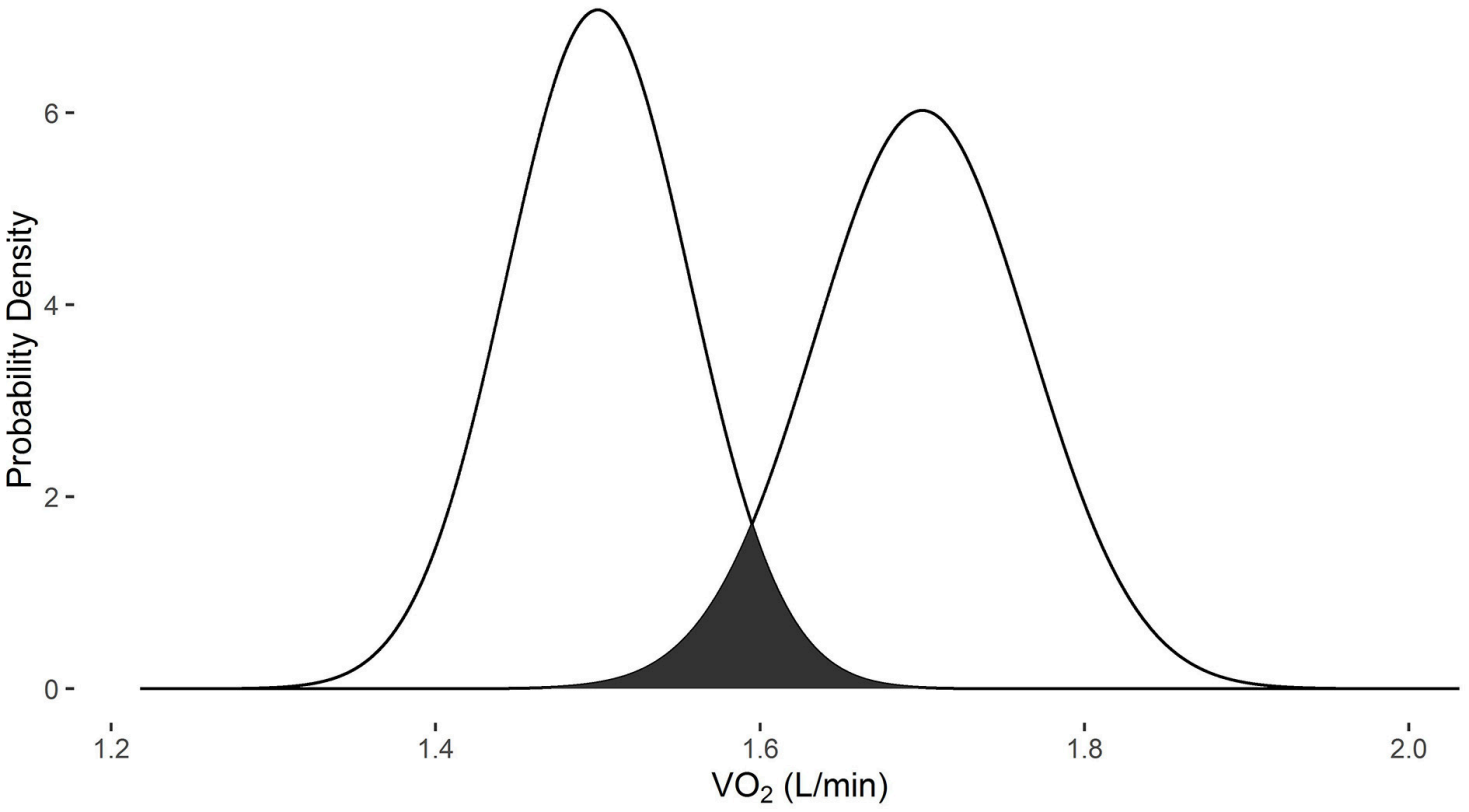
**Overlapping probability density plots for VO_2_ measures of 1.5 L/min and 1.7 L/min collected with the Parvomedics 2400 TrueOne system**. The dark overlapping section results in an overlapping coefficient of 0.103.

**Figure 2:**
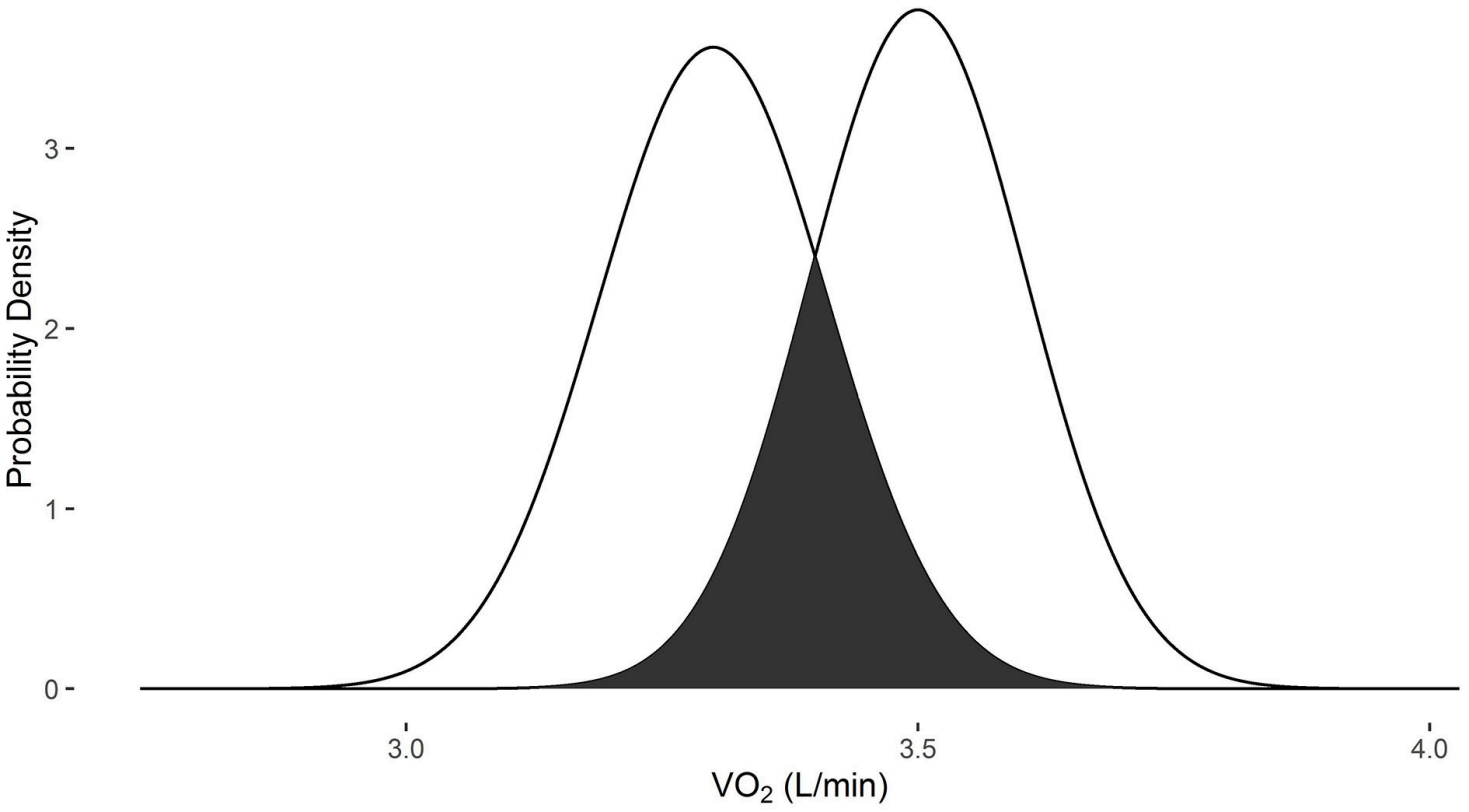
**Overlapping probability density plots for VO_2_ measures of 3.3 L/min and 3.5 L/min collected with the Parvomedics 2400 TrueOne system**. The dark overlapping section results in an overlapping coefficient of 0.358.

The change in VO_2_ after training protocols is an example of how VO2sim can be used to determine if repeated VO_2_ measurements are within the differential measurement error based on the specific system used to obtain the measurement. In examples 1 and 2, the measurements were obtained with the Parvomedics 2400 TrueOne system. When the baseline VO_2_ is 1.5 L/min and post-intervention VO_2_ is 1.7 L/min, there is a 10.3% probability that they are the same measure (Figure 1). When the baseline VO_2_ is 3.3 L/min and post-intervention VO_2_ is 3.5 L/min, there is a 35.8% probability that they are the same measure (Figure 2).

**Figure 3:**
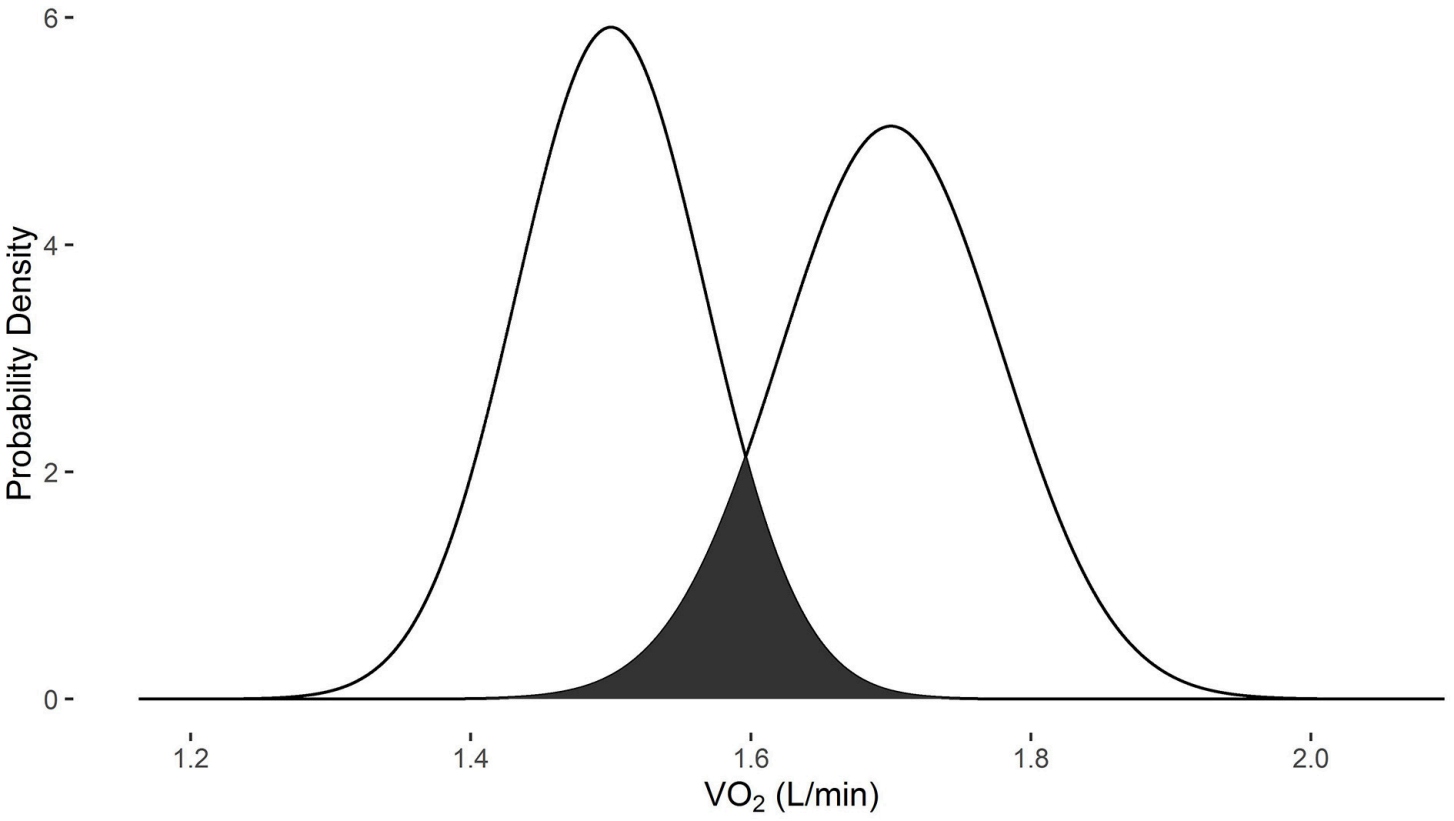
**Overlapping probability density plots for VO_2_ measures of 1.5 L/min and 1.7 L/min collected with the Douglas bag**. The dark overlapping section results in an overlapping coefficient of 0.172.

**Figure 4:**
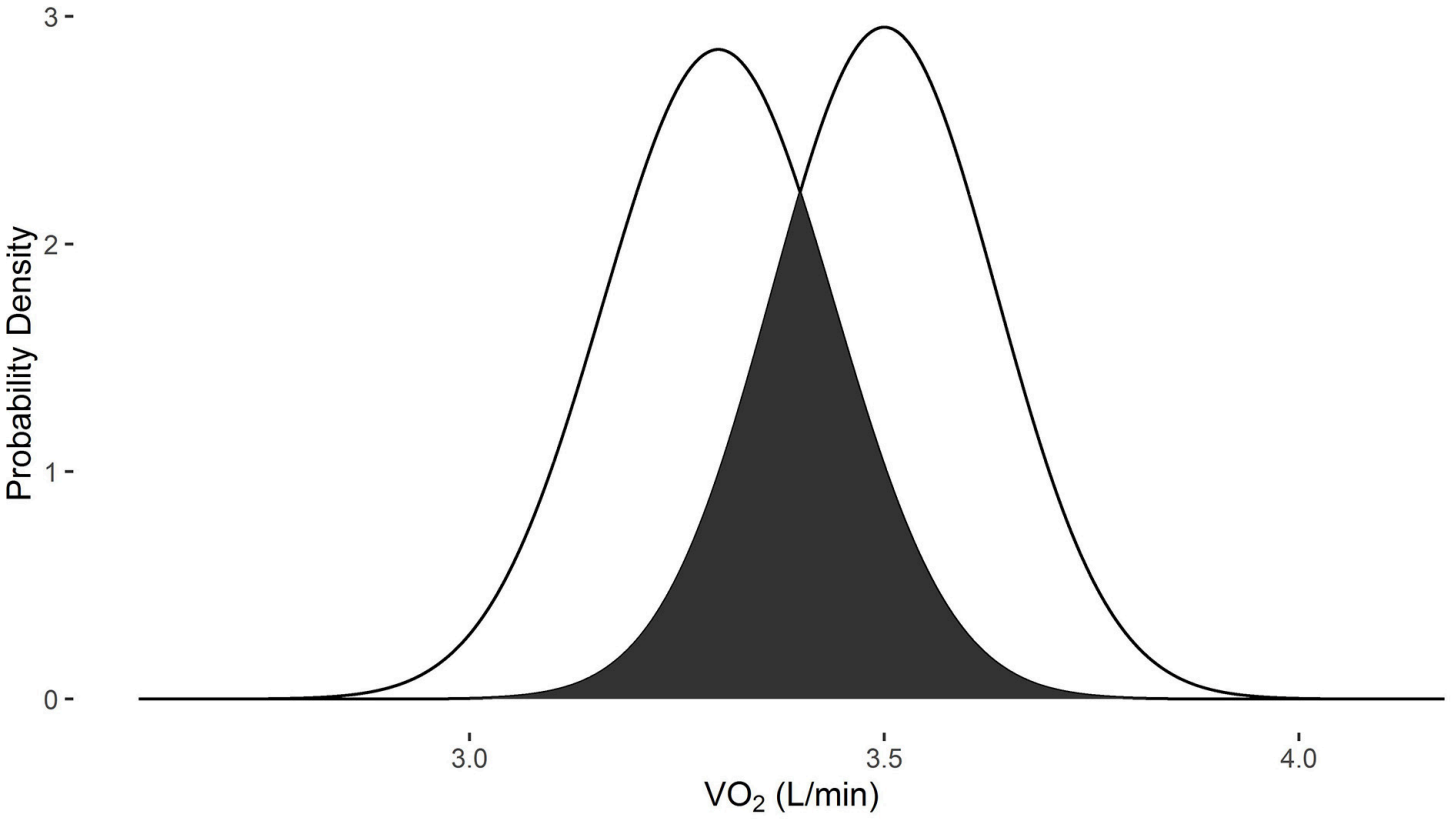
**Overlapping probability density plots for VO_2_ measures of 3.3 L/min and 3.5 L/min collected with the Douglas bag**. The dark overlapping section results in an overlapping coefficient of 0.467.

In examples 3 and 4, the measurements were obtained with the Douglas bag method. When the baseline VO_2_ is 1.5 L/min and post-intervention VO_2_ is 1.7 L/min, there is a 17.2% probability that they are the same measure (Figure 3). When the baseline VO_2_ is 3.3 L/min and post-intervention VO_2_ is 3.5 L/min, there is a 46.7% probability that they are the same measure (Figure 4).

**Figure 5:**
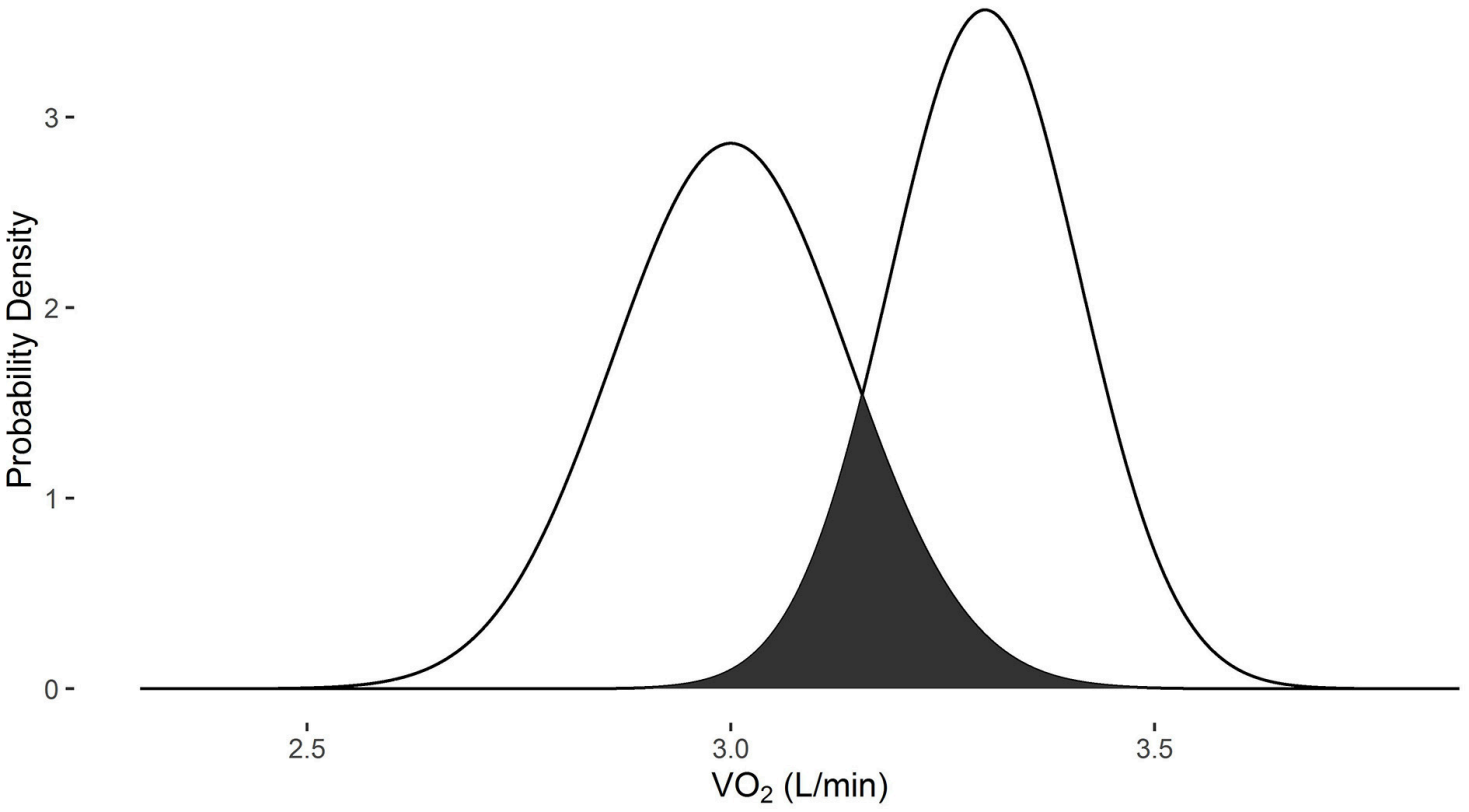
**Overlapping probability density plots for VO_2_ measures of 3.0 L/min collected with the Douglas bag and 3.3 L/min collected with the Parvomedics 2400 TrueOne system**. The dark overlapping section results in an overlapping coefficient of 0.231.

Example 5 demonstrates the use of VO2sim to compare VO_2_ measures when they are obtained from different systems. When the baseline VO_2_ of 3.0 L/min is obtained with the Douglas bag and the follow-up VO_2_ measurement of 3.3 L/min is obtained with the Parvomedics 2400 TrueOne system, there is a 23.1% probability that they are the same measure (Figure 5).

## Discussion

This study presents a novel descriptive methodology and tool to examine measurement error in gas exchange indirect calorimetry. This method is not susceptible to issues of statistical power, nor is it directly designed for any type of hypothesis testing. VO2sim adds an additional layer to ensure that clinical interpretations are valid as well as for didactic purposes within the classroom. To facilitate use by researchers and practitioners, this tool is available both as a statistical package within R and as a GUI.

### Graphical user interface

In recognition that there are a wide number of researchers and practitioners who may benefit from VO2sim but may not be comfortable with the command line interface used in R programming (a suitable introduction to R is “R in a Nutshell”; Adler, 2010), an online GUI has been implemented using Shiny Apps for R. This web application (https://tenan.shinyapps.io/VO2sim) enables easy input of test values and returns the probability that the two measures are the same. It also produces a visual representation of the distributions and amount of overlap. All images within the present manuscript can be re-created using the web application. The primary downside to the GUI implementation is that it is unable to be iteratively run on multiple participants, requires individual input and does not allow the user full access to the underlying graphics.

### Appropriate application of VO2sim as a tool

In cases where a single-subject analysis is performed, VO2sim can be used as the primary analytic tool. In research studies with multiple subjects, hypothesis testing should be performed prior to analysis with VO2sim. If VO_2_ normalized to body mass (typically, mL/kg/min) is desirable, normalized VO_2_ can be used in the hypothesis testing while the non-normalized VO_2_ data is used in the VO2sim analysis. If the hypothesis testing indicates that a statistically significant difference is observed between time points, VO2sim can be “stacked” or applied to each subject's data individually and the mean of the subjects' probability of similarity can be calculated to render a “net probability of similarity” between time points. The manual calculation of net probability of similarity with the VO2sim GUI can be time consuming depending on the number of subjects and also susceptible to human input error. However, when the net probability of similarity is calculated in the command line in R, this can be calculated using a single line of code:

$$\begin{array}{l} \text{mean}\left(\text{mapply}\left(\text{VO}2\text{\_sim},\text{a}=\text{pre\_vector},\text{b}=\text{post\_vector}\right)\right) \end{array}$$

In this example, where the default ParvoMedics 2400 TrueOne system is used, only the vectors or pre- and post-data collection points need to be supplied (pre_vector and post_vector, respectively). It is anticipated that as statistical methods for models of differential measurement error become more available and accepted (Newton et al., 2001; Imai and Yamamoto, 2010), that the coarse method of using VO2sim in “stacked” form will become obsolete for research purposes.

### Implications of VO2sim on existing literature

Since VO2sim needs to be applied on the raw data within a study, there are few published studies which can be directly evaluated. However, rough approximations of previous work can be performed based upon the reported mean values of VO_2_ and an assumed use of gold standard methodology (Douglas bag). The Gas.Sim tool may indicate the presence of a Type 1 statistical error, where there is an incorrect rejection of the null-hypothesis (i.e., “false positive”). In the present context, a Type 1 error may occur in small sample studies because VO_2_ measures within the error range happen to be obtained on one side of the distribution or in a larger sample study where there is statistical power to detect differences which exceed the accuracy of the device. This is especially likely in research when data is collected until findings are “significant” (Simmons et al., 2011). In practice, this is not a valid use of VO2sim, but it provides theoretical examples of how VO2sim can be used to assess measurement error in real-world data.

Variability and uncertainty is inherent in any testing methodology. Typically, devices with low measurement error can corroborate the findings of devices with higher measurement error. VO2sim is able to provide a context for the level of confidence in the VO_2_ metric apart from any corroborating data. For example, Lorenzo et al. (2010) recently demonstrated that heat acclimation improves exercise performance. In addition to a number of other physiologic variables, it is reported that mean VO_2_ in a 1 h cycling task increased by 5% in cool and 8% in hot conditions. Based on the sample mean data reported in the study, VO2sim returns a 68.6% and 27.3% probability that VO2 levels are the same after heat acclimatization in cool and hot conditions, respectively. Similarly, Howden et al. (Howden et al., 2015) indicated that females have a decreased training response to an endurance regimen with observable increases in VO_2max_ at 3, 6, 9, and 12 months (2.48, 2.57, 2.48, and 2.51 L/min, respectively) compared to baseline (2.19 L/min). According to VO2sim, this results in a similarity probability of 20.9, 10.5, 20.9, and 16.8% at 3, 6, 9, and 12 months, respectively. When considering the above examples it is important to note that these are assessments of the overall averages reported and not indicative of that study's individual data. Furthermore, the listed studies contain numerous other metrics supporting their conclusions that have greater accuracy than gas exchange indirect calorimetry. Nonetheless, it is important to understand the context and reliability of the reported VO_2_ results.

### Comparison with existing methodologies for single subject analysis

It is important to consider the Gas.Sim package and VO2sim function in relation to other methods proposed to understand differences in repeated VO_2_ measurements for singular subjects. The methods proposed by both Hopkins (2015) and Hecksteden et al. (2015) use a theoretical threshold by which a measurement or difference in measurements are clinically or practically meaningful. This should be a substantial consideration when evaluating the effectiveness of an intervention. VO2sim does not account for the magnitude required for clinical meaning. What defines practical significance is, in part, an opinion of the clinician, researcher, editor and/or journal reviewer (Riemann and Lininger, 2015). Indeed, even within researchers, the conception of what defines practical significance may change across a long period of time (Hopkins, 2004).

Probably the most meaningful differences between the methodologies of Hopkins (2015) and Hecksteden et al. (2015) and the Gas.Sim package are the ways in which the measurement error itself is considered. Both previous methods allow the researcher/clinician to dictate the standard error of measurement or coefficient of variation for their methodology. VO2sim dictates the differential measurement error based upon the system being used and flow volume. This represents a trade-off whereby each approach has certain advantages and disadvantages. Allowing the researcher/clinician to dictate the measurement error enables them to input error rates that may be more specific to their situation. For instance, the researcher/clinician may have previously performed a reliability analysis of their own specific machine and skilled implementation across a variety of gas flow rates, this will clearly better approximate the likely error than a published study from a different research laboratory. The downside to allowing manual input of error is that researchers/clinicians may over-estimate their personal level of skill or the error rate of the device. There may also be cases where error levels are manually adjusted (or different reliability studies used) until a result is rendered which satisfies the researcher/clinician. VO2sim does not allow researcher/clinicians to alter the reliability settings, except for indirect calorimetry device selection which, by definition, needs to be determined prior to data collection. VO2sim also adjusts the reliability of the indirect calorimetry device (i.e., accounts for differential measurement error) based upon the flow rate as both the data underlying VO2sim and other published works have indicated that system reliability fluctuates based on air flow (Crouter et al., 2006; Macfarlane and Wu, 2013). Generally, the methods by Hopkins and Hecksteden et al. suggest that this reliability stays constant for a given device; however, the researcher/clinician is able to change the reliability based on their flow rate if they have data supporting the reliability at that given flow. Overall, the Hopkins and Hecksteden et al. methods allow for greater researcher degrees of freedom than VO2sim, enabling (potentially) both more accurate reliability estimates for a specific situation as well as more investigator error and confirmation bias.

The Gas.Sim package has the benefit of returning a probability of similarity between two indirect calorimetry measures. This can be interpreted in a straightforward way: “there is a 30% probability that the two measures are the same.” A reasonable default threshold to state that the measures are “truly different” is 10% similarity. However, users of the Gas.Sim package are encouraged to consider what level of similarity is acceptable given their particular context.

### Present limitations and future modifications

The Gas.Sim package is presently limited to providing estimates for only two systems: ParvoMedics 2400 TrueOne and Douglas bag. As raw day-to-day validation data becomes available, new systems will be added to Gas.Sim's capabilities. The GUI is only available for VO2sim; however, the Gas.Sim package available for the R interface has functions capable of examining minute ventilation (VE) and carbon dioxide (VCO_2_). The current iteration of Gas.Sim is only valid for examination of day-to-day variability. This variability takes into account both the human-level variability and the system-level variability. Using VO2sim to determine the probability of VO_2_ differences within a testing session will likely result in an overly conservative estimate. As raw data becomes available which isolates the system-level variability, it will be added to the software package to estimate within-trial VO_2_ differences.

The Gas.Sim package relies heavily on the raw validation data provided by outside investigators (Crouter et al., 2006). As such, it assumes that the validation data was collected using best practices under “normal circumstances.” Therefore, estimates may be conservative if the underlying raw data did not maintain appropriate methodologies or standardization and may be overly liberal if the underlying raw data was collected under near-perfect circumstances which other investigators are unable to achieve. The raw data underlying the current version of Gas.Sim (Crouter et al., 2006) has been previously published in an established journal and was produced by a research group with a long-standing history of published validation work. This suggests a high-level of confidence in the raw data upon which Gas.Sim is based.

## Conclusions

Simulation of reliability data in gas exchange indirect calorimetry provides a method by which measurement error can be quantified and assessed. Both a command line and GUI implementation of the VO2sim function are presently available and described within the manuscript. Future iterations of the Gas.Sim package will include a greater number of indirect calorimetry devices as the raw validation data is made available. The described statistical tool provides an additional layer of security to understand and quantify the validity of clinical and research outcomes in exercise testing.

## Author contributions

MT developed the methodology in the present manuscript and wrote the manuscript and underlying code for both the software package and web application.

### Conflict of interest statement

The authors declare that the research was conducted in the absence of any commercial or financial relationships that could be construed as a potential conflict of interest.
